# Signs of Death

**Published:** 1874-10

**Authors:** 


### SIGNS OF DEATH

Put a live coal on the skin, if it blisters, there is life.

In looking at a colored thermometer sometimes, there will be seen an interruption in the column as if there was an "air bubble" between; in case of real death, it is said that if the eye is examined with an opthœmoscope the blood in the veins has this interrupted appearance.

